# New Delhi Metallo-β-Lactamase-1 in Carbapenem-Resistant *Salmonella* Strain, China

**DOI:** 10.3201/eid1912.130051

**Published:** 2013-12

**Authors:** Jinwei Huang, Minghua Wang, Hui Ding, Meiping Ye, Fupin Hu, Qinglan Guo, Xiaogang Xu, Minggui Wang

**Affiliations:** Huashan Hospital, Fudan University, Shanghai, China (J. Huang, M. Wang, F. Hu, Q. Guo, X. Xu, M. Wang);; Lishui Central Hospital, Lishui, China (J. Huang, H. Ding); and Wenzhou Medical University, Wenzhou, China (M. Ye)

**Keywords:** carbapenemase, New Delhi metallo-β-lactamase-1 NDM-1, Salmonella spp., bacteria, carbapenem, carbapenem resistance, antimicrobial resistance, plasmid-mediated, acute diarrhea, China

**To the Editor:** Carbapenem resistance in Enterobacteriaceae can occur through the production of carbapenem-hydrolyzing enzymes such as New Delhi metallo-β-lactamase-1 (NDM-1) (*1*). In recent years, plasmid-mediated NDM-1 has spread rapidly worldwide and into multiple Enterobacteriaceae species, such as *Klebsiella pneumoniae* and *Escherichia coli* (*2*).

NDM-1 has been reported in 2 strains of *Salmonella* spp., which were isolated from feces and urine specimens during screening for multidrug-resistant bacteria in patients from India (*3**,**4*). We report the isolation of 1 community-acquired NDM-1–bearing *Salmonella* strain isolated from a child with acute diarrhea.

The *Salmonella* strain was isolated from the feces of an 11-month-old girl at Lishui Central Hospital, Zhejiang Province, China, on July 25, 2012. Six days before admission, a fever ≤40°C, accompanied by a cough, developed in the patient. Four days before admission, physical examination showed fine rales in both lungs. The leukocyte count was 8,900 cells/µL, with 80% neutrophils. No obvious abnormalities were found on a chest radiograph.

The patient was given a diagnosis of acute bronchitis, and the condition was treated with parenteral cefoxitin for 3 days and parenteral piperacillin/tazobactam for 1 day, but fever persisted. Two days before admission, diarrhea (4–5 times/day with loose feces containing mucus and blood) developed. On admission day, fecal analysis showed 3–4 leukocytes and 1–3 erythrocytes per high-power field. A *Salmonella* sp. was isolated from feces obtained at admission and identified as *S. enterica* subsp. *enterica* serovar Stanley by serotyping by the local Centers for Disease Control and Prevention.

The patient was then given a diagnosis of bacterial enteritis and received intravenous azithromycin and latamoxef. Fever and diarrhea resolved over the next 3 days. On the fifth day of hospitalization, a fecal culture was negative for *Salmonella* spp. and the patient was discharged. At a follow-up visit 3 months later, *Salmonella* spp. or other carbapenem-resistant bacteria were not isolated from feces samples from the patient or her grandmother and brother, who lived with her.

The patient and her family had not traveled to any country during the year, including countries with a high prevalence of NDM-1 producers. The patient was living in a small rural village in southern China and did not have a special diet. She was healthy before hospitalization for fever. She was born by cesarean section and did not have contacts with hospitalized patients.

MICs of antimicrobial drugs were determined by agar dilution and interpreted by using revised Clinical and Laboratory Standards Institute breakpoints (*5*). The *Salmonella* Stanley strain was resistant to all β-lactam antimicrobial drugs tested, including cephalosporins and carbapenems, but susceptible to chloramphenicol, ciprofloxacin, tetracycline, and fosfomycin, and had azithromycin MICs of 4 µg/mL (Table).

**Table:** Antimicrobial drug susceptibility of *Salmonella* strain Stanley and transconjugants containing New Delhi metallo-β-lactamase-1, China

Drug	MIC, μg/mL

*Salmonella* strain Stanley	*Escherichia coli* C600	*E. coli* C600 transconjugant	*Klebsiella pneumoniae* 13883	*K. pneumoniae* 13883 transconjugant
Piperacillin//tazobactam	>128/4	2/4	128/4	1/4	128/4
Ceftazidime	>128	0.25	>128	0.25	>128
Cefotaxime	>128	<0.06	128	<0.06	>128
Latamoxef	64	0.25	32	<0.06	32
Cefepime	16	<0.06	16	<0.06	16
Imipenem	8	<0.06	8	<0.06	16
Meropenem	4	<0.06	4	<0.06	8
Fosfomycin	<0.06	<0.06	<0.06	<0.06	<0.06
Minocycline	2	0.5	0.5	1	0.5
Ciprofloxacin	<0.06	<0.06	<0.06	<0.06	<0.06
Chloramphenicol	2	4	4	4	4
Azithromycin	4	1	1	2	1
Trimethoprim/sulfamethoxazole	>152/8	0.3/0.015	>152/8	1.2/0.06	>152/8

A modified Hodge test result for *Salmonella* strain Stanley was weakly positive. Production of metallo-β-lactamase was detected by using an imipenem-EDTA double-disk synergy test. Carbapenamase-encoding genes, including *bla*_KPC-2_, *bla*_VIM_, *bla*_IMP_, *bla*_SPM-1_, *bla*_GIM-1_, and *bla*_SIM-1_, were detected by using PCR as described (*2*). Only the *bla*_NDM-1_ gene was detected (with primers 5′-GGCGGAATGGCTCATCACGA-3′ and 5′-CGCAACACAGCCTGACTTTC-3′). The PCR product sequence was consistent with that of NDM-1 (GenBank accession no. FN396876).

Conjugation experiments were conducted as described (*6*). Carbapenem resistance could be transferred from *Salmonella* strain Stanley to *E. coli* C600 Rif^r^ and *K. pneumoniae* 13883 Rif^r^ at frequencies of 1 transconjugant per ≈1.0 × 10^4^ and 4.0 × 10^7^ bacterial cells, respectively, after exposure for 15 min.

Plasmid DNA was extracted by using a Plasmid Midi Kit (QIAGEN, Hilden, Germany) according to the manufacturer’s instructions. Electrophoresis showed that donor and transconjugant strains had the same plasmid profile; both contained an ≈140-kb plasmid. A PCR-based method for plasmid replicon typing (*7*) indicated that the plasmid belonged to incompatibility group IncA/C.

Experiments of *bla*_NDM-1_ stability in *Salmonella* spp., *E. coli* transconjugants, and *K. pneumoniae* transconjugants were conducted by using the method of Wang et al. (*6*). Twenty colonies were randomly selected every day during days 2–15. Only 2 colonies of the *Salmonella* Stanley strain lost carbapenem resistance; these colonies were collected on the second and ninth days of passage, respectively. Plasmids containing *bla*_NDM-1_were not present in these 2 colonies. No transconjugants lost carbapenem resistance during 14 days of passage.

Although *Salmonella* spp. have shown increased resistance to cephalosporins and quinolones, resistance to carbapenems is rare (*3**,**4**,**8**,**9*). Because of emerging resistance to traditionally recommended antimicrobial agents, azithromycin is increasingly used for treatment of invasive *Salmonella* spp. infections in children (*10*). The patient with carbapenem-resistant *Salmonella* infection and acute diarrhea was cured by treatment with azithromycin.

This report indicates ongoing spread of NDM-1–bearing *Salmonella* strains. If one considers the high conjugation frequency and stability of the IncA/C plasmid containing NDM-1 in *Salmonella* spp., one would conclude that it might increase spread of bacterial drug resistance. Prompt recognition of carbapenem-resistant *Salmonella* spp. and initiation of appropriate infection control measures are essential to avoid spread of these organisms.
